# The role of nucleoside triphosphate hydrolase metallochaperones in making metalloenzymes

**DOI:** 10.1093/mtomcs/mfac030

**Published:** 2022-04-29

**Authors:** Francesca A Vaccaro, Catherine L Drennan

**Affiliations:** Department of Chemistry, Massachusetts Institute of Technology, 77 Massachusetts Avenue, Cambridge, MA, USA; Department of Chemistry, Massachusetts Institute of Technology, 77 Massachusetts Avenue, Cambridge, MA, USA; Department of Biology, Massachusetts Institute of Technology, 77 Massachusetts Avenue, Cambridge, MA, USA; Howard Hughes Medical Institute, Massachusetts Institute of Technology, Cambridge, MA, USA

**Keywords:** metallochaperones, GTPases, ATPases, metalloenzyme maturation, metallocofactors, metals in biology

## Abstract

Metalloenzymes catalyze a diverse set of challenging chemical reactions that are essential for life. These metalloenzymes rely on a wide range of metallocofactors, from single metal ions to complicated metallic clusters. Incorporation of metal ions and metallocofactors into apo-proteins often requires the assistance of proteins known as metallochaperones. Nucleoside triphosphate hydrolases (NTPases) are one important class of metallochaperones and are found widely distributed throughout the domains of life. These proteins use the binding and hydrolysis of nucleoside triphosphates, either adenosine triphosphate or guanosine triphosphate, to carry out highly specific and regulated roles in the process of metalloenzyme maturation. Here, we review recent literature on NTPase metallochaperones and describe the current mechanistic proposals and available structural data. By using representative examples from each type of NTPase, we also illustrate the challenges in studying these complicated systems. We highlight open questions in the field and suggest future directions. This minireview is part of a special collection of articles in memory of Professor Deborah Zamble, a leader in the field of nickel biochemistry.

## Graphical Abstract

**Graphical Abstract:**
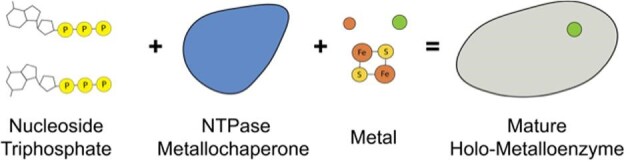
Nucleoside triphosphates are hydrolyzed by metallochaperones (blue) to insert metal ions, multi-metal clusters, or organometallic clusters to form the matured metalloenzyme (gray).

## Introduction

**Fig. 1:**
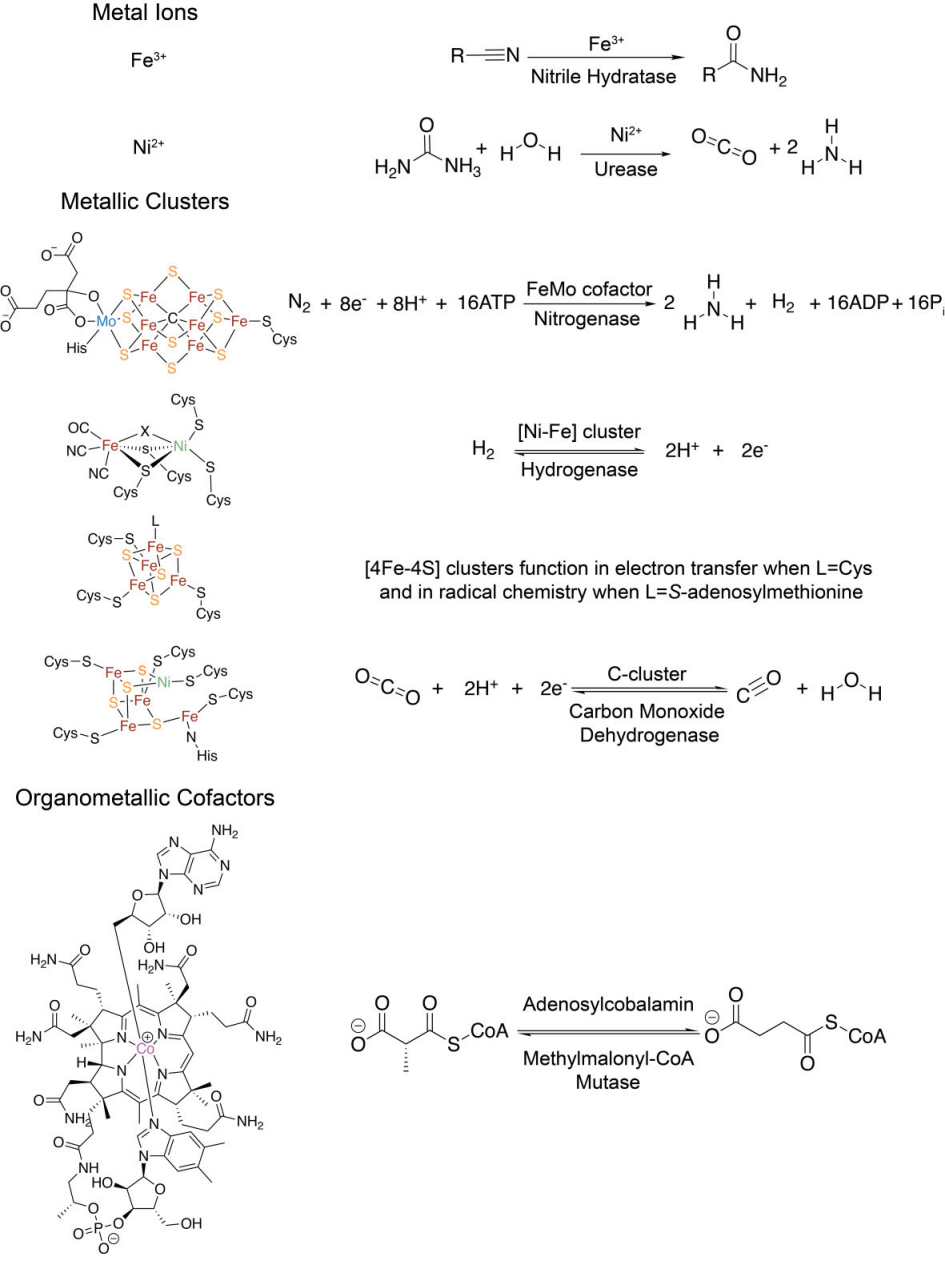
Metallocofactors vary widely in structure and reactivity. In some reactions, metal ions such as Fe^3+^ or Ni^2+^ are ligated by residues of the metalloprotein but still require metallochaperones for proper maturation. Increasingly complex metallic clusters, such as the FeMo-cofactor and [4Fe–4S] clusters, are transported and inserted by metallochaperones, whereas other metallic clusters, such as the [NiFe] cluster, are assembled *in situ*. Additionally, organometallic cofactors are often metabolically expensive and are transported by metallochaperones into their target metalloenzyme to perform difficult chemistry.

Metal sites exist in about one-third of all structurally characterized enzymes, and over half of all proteins are predicted to be metalloproteins.^1^ These metal sites range in complexity from one metal ion to multi-metal or even organometallic clusters (Fig. 1).^2^ Metalloenzymes tend to catalyze the most challenging chemical transformations. Their roles include, but are not limited to, respiration^3^; photosynthesis^4^; regulation of transcription and translation^3^; and nitrogen,^5^ carbon,^6^ and hydrogen fixation.^7^ To have a functioning metalloenzyme, the metallocofactor must be correctly installed, which often requires a metallochaperone. Metallochaperones are proteins that physically interact with apo-metalloprotein clients or intermediary proteins to assist in cofactor delivery or assembly as part of the process of metalloprotein maturation. The exact percentage of metalloproteins that require metallochaperones for cofactor biogenesis is not established. Quantification is complicated by the fact that metallochaperones are often not needed for metalloprotein reconstitution in vitro but are in vivo, where metal ion concentrations are often limiting due to toxicity issues. In the cell, metallochaperones balance the cellular demand for metal-assisted reactivity with the toxicity associated with having too much metal.

Deletion or mutation of genes encoding metallochaperones can lead to deleterious biological consequences. In *Azobacter vinelandii*, deletion of the metallochaperones NifX and NafY decreases the amount of active nitrogenase by half, reducing levels of dinitrogen reduction and stunting organismal growth.^9,10^ In humans, the metabolic disorder methylmalonic aciduria is caused by mutations to or deletion of the gene for methylmalonic aciduria type A protein (MMAA), a metallochaperone required for the maturation of adenosylcobalamin-dependent methylmalonyl-CoA mutase (MCM). Without the adenosylcobalamin cofactor, methylmalonyl-CoA cannot be converted into succinyl-CoA, causing an accumulation of methylmalonic acid that alters the blood pH leading to disease.^11,12^ Because of the deleterious effects of metallochaperone impairment, bacterial metallochaperones are also potential drug targets. For example, the metallochaperone UreG is necessary for the maturation of the dinickel metal active site of urease, an enzyme that catalyzes the hydrolysis of urea to ultimately yield ammonia and carbon dioxide, providing the buffering capacity necessary for *Heliobacter pylori* to live in the stomach. As such, urease is central to *H. pylori* metabolism and virulence,^13,14^ making its metallochaperones potential drug targets.

The wide variety of metal centers employed by metalloproteins requires a diversity of metallochaperones. There are numerous types of metallochaperones that have been characterized, from the transporters of the FeMo—cofactor of nitrogenase^15,16^ to the copper metallochaperones involved in shuttling copper ions to the mitochondrial electron transport chain for cellular respiration.^17^ This minireview focuses on one group of metallochaperones: nucleoside triphosphate hydrolase (NTPase) metallochaperones. NTPase metallochaperones use the binding and/or hydrolysis of nucleoside triphosphates (either GTP or ATP) for metalloenzyme maturation. They exist widely in the domains of life,^18^ and although they are ubiquitous, their exact roles in maturation processes are often not well understood. Known and postulated roles include: metal ion or metallocofactor transport to an intermediary protein in the metalloprotein maturation process; metallocofactor binding and direct insertion into apo-target protein; and induction of a conformational change in the target protein to allow for cofactor delivery. Here, we focus on the recent advances in the study of NTPase metallochaperones, describing the current state of knowledge and highlighting open questions.

## P-loop G3E GTPase metallochaperones

The first subclass of NTPase metallochaperones that we will consider are phosphate-binding loop (P-loop)-containing G3E GTPases (hydrolyzing guanosine triphosphate, GTP).^18^ The P-loop G3E GTPase family is named for a D-to-E substitution in the G3 motif compared to the canonical P-loop GTPase, Ras.^18^ The best characterized subfamilies of the P-loop G3E GTPase metallochaperone family are the urease metallochaperone UreG,^19,20^ the [NiFe]-hydrogenase metallochaperone HypB,^21^ and the cobalamin metallochaperone MeaB (MMAA in humans) subfamilies.^22–24^ In addition to UreG, HypB, and MeaB/MMAA, there is a large and diverse subfamily of G3E GTPases called the cluster of orthologous groups (COG) 0523 proteins (Table 1). COG0523 proteins are characterized by a conserved CxCC (C = Cys; x = any amino acid) motif that is implicated in high affinity metal binding.^25,26^ Known functions of COG0523 proteins include: zinc homeostasis (ZigA/ZagA);^27^ cobalamin cofactor biosynthesis (CobW);^28–30^ and nitrile hydratase maturation (Nha3).^31^

**Table 1:** Subfamilies of the G3E P-Loop GTPases

Subfamily	Known roles	Known metals/metallocofactor
UreG	Maturation of ureases	Nickel
HypB	Maturation of [NiFe]-hydrogenases	Nickel
MeaB	Maturation of adenosylcobalamin-dependent mutases	Cobalamin
COG0523	Zinc homeostasis (ZigA/ZagA), Nitrile hydratase maturation (Nha3), Cobalamin cofactor biosynthesis (CobW)	Zinc Iron Cobalt

## P-loop G3E GTPases employ a common protein fold

**Fig. 2:**
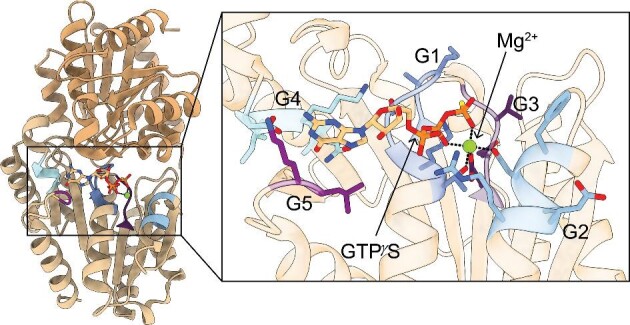
Dimeric structure of P-loop-containing G3E GTPase metallochaperone with conserved motifs. (Left) Representative structure: HypB from *Methanocaldococcus jannaschii* (PDB 2HF8) with guanosine 5′-O-(3-thiotriphosphate) (GTPγS) and Mg^2+^ bound.^37^ Structure is a dimer with each monomer containing a G-domain. (Insert) The GTP-binding site contains the G1–G5 motifs, which are highlighted. The G1 residues (dark blue), also known as the Walker A motif, interact with the α and β phosphates and the bound Mg^2+^ ion. The G2 residues (light blue), also known as switch I, change conformation based on the nucleotide state and the conserved aspartate residue coordinates the bound Mg^2+^ ion. The G3 residues (dark purple), also known as the Walker B motif, coordinate the γ-phosphate and the bound Mg^2+^ ion. The G4 residues (cyan) confer nucleotide specificity as they interact with the base of the nucleotide. The G5 residues (light purple) are involved in nucleotide dissociation.

All the known structures of the G3E P-loop GTPases contain a G-domain (Table 2) that is comprised of a very common protein fold^32^: regularly recurring α–β units with the β strands forming a central β-sheet surrounded on both sides by α-helices (Fig. 2). The typical G-domain contains five different conserved motifs, known as G1–G5, that are involved in nucleotide binding and hydrolysis (Fig. 2). The G1 motif, also known as the Walker A motif, is a flexible loop in between a helix and a sheet. This motif functions to position the triphosphate of the bound nucleotide. The G2 motif, also known as switch I, signals which nucleotide, if any, is bound. The G3 motif, also known as the Walker B motif loop, is often situated at the end of a strand and contains a conserved aspartate or, less commonly, glutamate residue that binds the water-bridged Mg^2+^ ion used for NTP hydrolysis.^32^ The G4 motif typically contains the sequence motif NKXD that is used in the recognition of the guanosine base.^18^ Finally, the G5 motif is involved in nucleotide release.^32^ The ubiquitous nature of P-loop NTPases has led to further classification of various structural motifs that are beyond the scope of this review but have been previously detailed by Leipe et al.^18^

**Table 2:** Example structures of members of the G3E P-loop GTPases

Subfamily	Protein	Organism	PDB IDs: ligand(s) bound
MeaB	MeaB	*Methylobacterium extorquens*	2QM8: no ligands bound^33^ 2QM7: GDP^a^ bound^33^ 4JYB: GMPPNP^b^ bound^34^
	MMAA	*Homo sapiens*	2WWW: GDP bound^35^
	MeaB fusion protein (IcmF)	*Cupriavidus metallidurans*	4XC7: no ligands bound^36^ 4XC8: GDP and Mg^2+^ bound^36^
HypB	HypB	*Methanocaldococcus jannaschii*	2HF9: GTPγS^c^ and Mg^2+^ bound^37^
		*Heliobacter pylori*	4LPS: GDP, Mg^2+^, Ni^2+^ bound^38^
UreG	UreG	*Klebsiella pneumoniae*	5XKT: GMPPNP, Ni^2+^ bound^39^
	UreG/UreF/UreH	*Helicobacter pylori*	4HI0: GDP bound^14^
COG0523	YjiA	*Escherichia coli*	4IXM: Zn^2+^ bound^26^

^a^GDP: guanosine diphosphate.

^b^GMPPNP: Guanosine 5′-[β,γ-imido]triphosphate.

^c^GTPγS: guanosine 5′-O-(3-thiotriphosphate).

## The mechanism of GTP hydrolysis is well established

The mechanism of nucleoside triphosphate hydrolysis by various GTPases has been extensively studied.^32,40–43^ Briefly, after nucleotide triphosphate binding, the active site is completed by a Mg^2+^-bound water molecule positioned by an aspartate (or less commonly, glutamate) to perform the hydrolysis of the terminal phosphate moiety of the nucleoside triphosphate. After the cleavage of the bond to the γ phosphate, the resulting nucleotide is a diphosphate; when replaced with a nucleoside triphosphate, the cycle can proceed again. Oftentimes, this binding and/or cleavage occurs in the presence of another protein known as an activating factor that increases the intrinsic rate of hydrolysis of the GTPase. In the case of metalloprotein maturation, the activating factor can be the target enzyme or another protein involved in the maturation process. Similarly, a nucleotide exchange factor is sometimes needed to replace the nucleoside diphosphate with the corresponding triphosphate.^32^ The changes throughout this cycle form the basis for the ability of the GTPases to perform their chaperone roles in the maturation of metalloproteinases.

## The well-studied family members UreG, HypB, and MeaB show mechanistic diversity

**Fig. 3:**
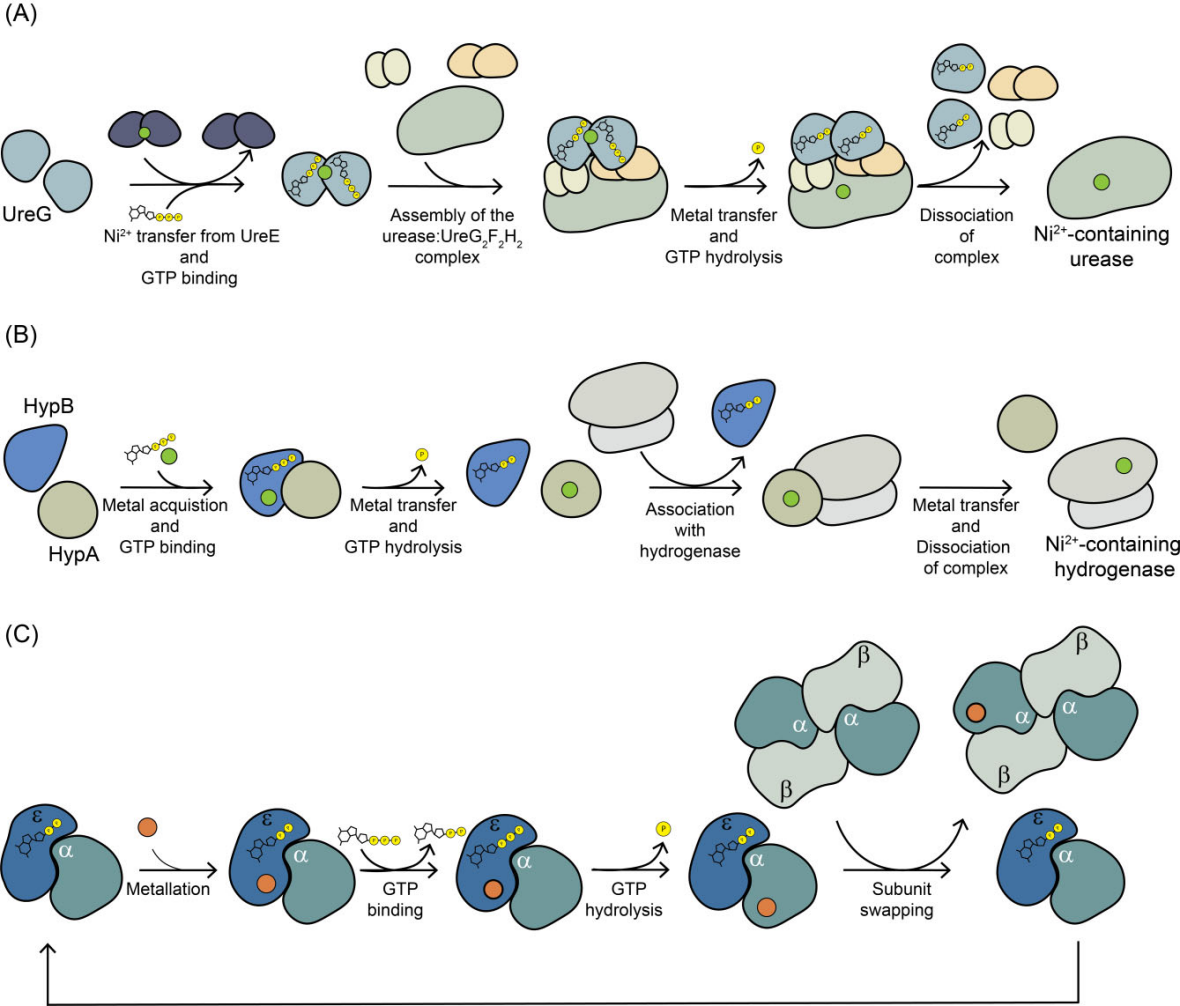
Postulated roles of G3E P-loop GTPases in metalloenzyme maturation. (A) Urease (pale green) requires the GTPase metallochaperone UreG (light blue) for insertion of the required Ni^2+^ ion. UreG receives the Ni^2+^ ion from UreE (dark blue) and binds GTP. The GTP binding event allows for the assembly of the apo-urease: the UreG_2_F_2_H_2_ complex. GTP hydrolysis triggers a conformational change that facilitates metal transfer to urease. After GTP hydrolysis occurs, the complex dissociates, and the urease now contains the needed Ni^2+^ ion for a fully activated enzyme.^14,19,39^ (B) [NiFe]-hydrogenases (light gray) require the metallochaperone HypB (blue) for Ni^2+^ ion insertion. HypB binds GTP and acquires Ni^2+^, which is transferred to HypA (pale green). HypA then interacts with the inactive hydrogenase to transfer the metal to the active site of the [NiFe]-hydrogenases for activation.^21,44,45,93,99^ (C) Fe-type NHases maturation with Fe ions (orange)^51,52^ involves a COG0532 GTPase metallochaperone, called ε or Nha3 (blue), which forms a complex with the α subunit of NHase (gray). The holo α subunit of NHase is swapped for an apo α subunit of NHase from the NHase α_2_ β_2_ complex. The process is repeated to fully mature the Fe-type NHases using the activating protein Nha3.^51^

The most well-studied family members of the G3E P-loop GTPase metallochaperones utilize different mechanisms for maturing their respective metalloenzymes despite employing a common, and potentially ancient protein fold.^18^ As far as we know, the evolutionary factors that drove these mechanistic differences within this metallochaperone subfamily are not understood. These mechanistic differences are especially interesting given that both UreG and HypB are metallochaperones involved in the maturation of the nickel-dependent enzymes: urease and [NiFe]-hydrogenase, respectively. UreG undergoes a conformational change when it binds GTP and accepts Ni^2+^ from UreE. Then, UreG forms a complex with apo-urease and the other accessory factors UreF, UreH, and UreD to directly insert the Ni^2+^ into its target enzyme, urease, through a tunnel created by the complex of all the accessory factors (Fig. 3A).^14,39,44^ Although HypB is also conformationally gated by GTP binding and hydrolysis, it does not directly insert Ni^2+^ into the [NiFe]-hydrogenase active site. The Zamble Lab showed that it is the GDP-loaded state of *Escherichia coli* HypB that is “readied” for fast Ni^2+^ transfer to accessory protein HypA, which ultimately leads to Ni^2+^ insertion into the active site of [NiFe]-hydrogenase (Fig. 3B).^45^ However, not all GTPase metallochaperones directly bind the metallocofactor that they are involved in delivering. In the maturation of methylmalonyl-CoA mutase, the GTPase metallochaperone MeaB (in bacterial systems, MMAA in humans) is required for adenosylcobalamin delivery; however, there is no evidence of the chaperone interacting with the cofactor itself.^24,35,46–49^

## COG0523 subfamily and its best characterized member Nha3

Giedroc and coworkers recently provided a comprehensive comparative phylogenetic, biochemical, structural, and functional analysis of P-loop G3E GTPases in the COG0523 subgroup.^50^ These proteins typically have two domains, a conserved G-domain at the N-terminus and a variable C-terminal domain that is predicted to be involved in target-specific protein interactions. The sequence analysis by Giedroc *et al*. suggests that there are multiple subfamilies within the COG0523 subgroup and that there is a considerable amount of unexplored sequence space. Many of the COG0523 sequence clusters have no characterized members. The lack of functional data is especially notable for COG0523 proteins from eukaryotic organisms. The best characterized C0G053 member is Nha3, a Fe-type nitrile hydratase (NHase) maturase. Currently, it serves as the model system for this diverse GTPase metallochaperone protein family.

Although Co-type NHases do not appear to require a GTPase metallochaperone,^31^ Fe-type NHases do. NHases catalyze the hydrolysis of organic nitrile to the corresponding amide product (Fig. 1) by using a metal ion (Co^3+^ or Fe^3+^) that is buried at the interface between the α and β subunits.^51,52^ The maturation of Co-type nitrile hydratases relies on an α subunit swapping mechanism that requires a GTP-independent maturation protein,^31^ whereas the maturation protein for Fe-type NHases is a COG0532 GTPase metallochaperone (called ε or Nha3) (Fig. 3C). Briefly, the swapping mechanism for the Fe-type NHases appears to involve a Fe^2+^-loaded GTP-bound Nha3 forming a complex with an apo α subunit of Fe-type NHase.^51,52^ In a GTP-dependent manner, the α subunit receives the metal and is oxidized to Fe^3+^ in the α subunit. Consistent with other G3E P-loop GTPases, the presence of divalent metals bound increases the rate of GTP hydrolysis of Nha3.^51^ The apo complex of the α_2_β_2_ NHase swaps an apo-α subunit with a holo-α subunit to form the holo-NHase.^51^ Substitutions of the conserved Lys or Thr of the P-loop of Nha3 from *Pseudomonas chlororaphis B23* result in an *in vivo* loss of detectable NHase activity similar to what has been observed for UreG and urease and HypB and [NiFe]-hydrogenase, further implicating the GTPase activity of Nha3 in Fe-type NHase maturation.^53^

## ATPase metallotransporters and metallochaperones

**Fig. 4:**
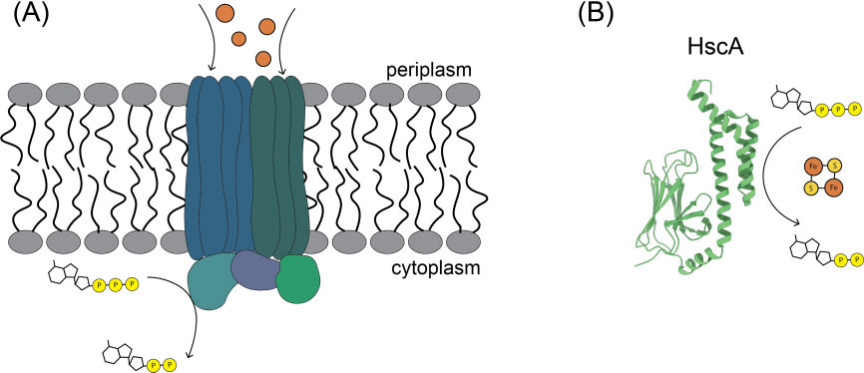
Some representative ATPases involved in metalloprotein maturation. (A) P1B-type ATPases are involved in the transport of heavy metals (orange spheres) into the cells to maintain metal homeostasis. The protein contains two domains: a transmembrane domain and a cytoplasmic ATPase domain. The flux of heavy metals is coupled to the hydrolysis of ATP.^54^ (B) The heat shock protein fold ATPases, such as HscA from E. coli (PDB 1U00), interact with co-chaperones in a nucleotide-dependent manner to transfer newly synthesized [2Fe-2S] clusters in the ISC biogenesis pathway.^100^

Compared to GTPases, there is a wider variety in the types of characterized ATPases (hydrolysis of adenosine triphosphate, ATP) involved in metalloprotein maturation (Fig. 4, Table 3).^54,55^ The P1B class of P-type ATPases uses the energy of ATP hydrolysis to transport d-block metal ions across cell membranes. In plants, the P1B class of P-type ATPases are key components in the maintenance of metal homeostasis, with characterized transporters for Zn^2+^, Cu^2+^, Cd^2+^, Co^2+^, and Pb^2+^ ions.^54^ These transporters serve as the first step in metalloprotein maturation by acquiring d-block metal ions from the surrounding environment. Analogous to the P-loop GTPases described above, P-loop ATPases are also involved in transporting metal ions to the active site of their target protein. One well-characterized P-loop ATPase metallochaperone is CooC,^56^ which is involved in the maturation of carbon monoxide dehydrogenases (CODHs).^57^ CODHs catalyze the reversible oxidation of carbon monoxide at a metallocofactor, the C-cluster, which consists of Ni, Fe, and S (Fig. 1). CooC is responsible for ATP-dependent Ni^2+^ insertion as part of the process of C-cluster maturation.^56–58^ Additionally, ATPases containing a heat shock protein fold are known to be involved in the biogenesis and transport of various metallic clusters. For example, in the iron-sulfur cluster (ISC) pathway found in mammals, the ATPase HscA works with the co-chaperone HscB to stimulate the transfer of nascently synthesized [2Fe–2S] to various apo enzymes in an ATP-dependent manner.^55,59^

**Table 3:** Subtypes of ATPase metallochaperones

Subtype	Known roles	Known metals/metallocofactor
P1B class	Transporting metal ions across membranes	d-block metals
P-loop	Maturation of metalloenzyme active sites	Ni^2+^
Heat shock fold proteins	Biogenesis of Fe–S clusters	Fe–S clusters

## Structures of P-loop ATPase CooC are similar to P-loop GTPase HypB

**Fig. 5:**
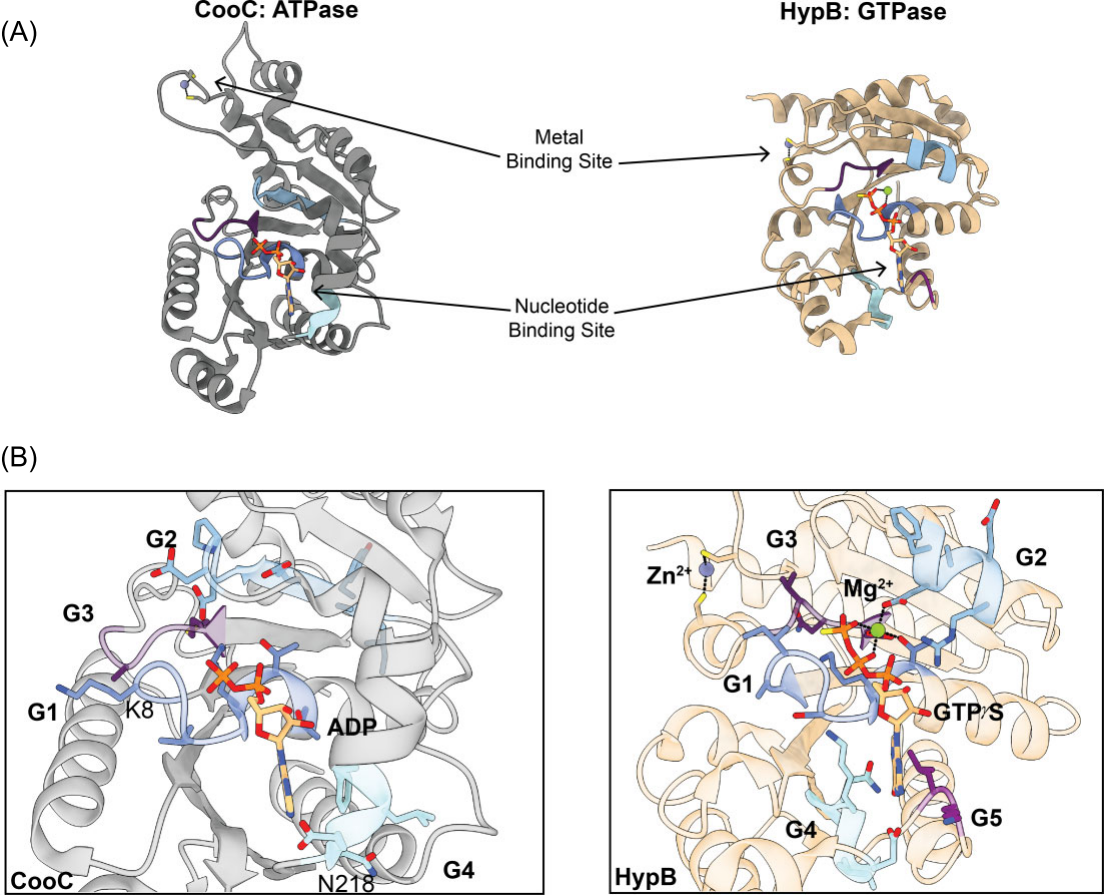
Comparison of the ATPase CooC and the GTPase HypB. (A) The ATPase metallochaperone CooC from *C. hydrogenoformans* (PBD 3KJI) (left, gray)^60^ and the GTPase metallochaperone HypB from *M. jannaschii* (PBD 2HF8) (right, tan)^37^ contain the conserved overall fold found in P-loop NTPases consisting of repeating α–β units. The metal-binding site and the nucleotide-binding site are spatially separated but involved in controlling the oligomeric states of the NTPases. Only one monomer for each metallochaperone is shown for simplicity. (B) (Left) A closer view of the ATP-binding site of CooC bound to ADP indicates that it contains the conserved G1–G3 motifs implicated in interacting with the phosphates of the nucleotide and the Mg^2+^ ion if present. The G1 residues (dark blue), also known as the Walker A motif, interact with the α and β phosphates and the bound Mg^2+^ ion. CooC contains the deviant Walker A motif with a highly conserved lysine residue (K8 in CooC) in the second position of the sequence instead of the second to last position. The G2 residues (light blue), also known as switch I, change conformation based on the nucleotide state and the conserved aspartate residue coordinates the bound Mg^2+^ ion. The G3 residues (dark purple), also known as the Walker B motif, coordinate the γ-phosphate and the Mg^2+^ ion if present. CooC only retains the first asparagine residue (N218 in CooC) of the G4 motif (cyan) that confers nucleotide specificity. An alpha helix, which is only present when a nucleotide is bound, interacts with the adenine base. (Right) A closer view of the GTP-binding site of HypB bound to GTPγS and a Mg^2+^ ion reveals the canonical G1–G5 motifs described in Fig. 2. Unlike CooC, HypB contains the conserved G4 motif (cyan) that confers nucleotide specificity by interacting with the guanosine base. The G5 residues (light purple) are involved in nucleotide dissociation and are not conserved in CooC.

Comparisons of the structures of CooC, a P-loop ATPase, and HypB, a P-loop GTPase, reveal an incredibly similar overall architecture of α–β units, despite low sequence homology of around 25% (Fig. 5A). Both proteins contain the G1–G3 sequence motifs that are involved in binding the phosphates of the nucleotide and the Mg^2+^ ion, although CooC contains the deviant G1 motif with the highly conserved lysine.^60^ The deviant G1 motif has the highly conserved residue of a typical G1 motif in the second position instead of the second-to-last position in the sequence motif, which is a characteristic of the MinD/BioD class of P-loop NTPases.^18^ The G4 nucleotide specificity motif in CooC is different from the conserved NKXD motif of HypB in that it only retains the first asparagine, and the CooC adenine base interacts with the residues of an alpha helix that is ordered upon binding the nucleotide (Fig. 5B).^60^ Additionally, these two proteins both have spatially separated but nucleotide-coupled metal-binding sites that control the oligomeric state of the NTPases (Fig. 5A).^60^

## Fe–S cluster biogenesis utilizes ATPases

Fe–S cluster biogenesis is a highly conserved yet complicated process that involves dedicated machinery to synthesize, transport, and deliver Fe–S clusters. This dedicated machinery involves proteins that act as chaperones and/or “scaffolds” for the assembly and delivery of Fe–S clusters. Notably, many of these proteins have ATP-binding sites and/or ATPase activity. There are four main Fe–S cluster biogenesis pathways: the sulfur fixation (SUF), the ISC, the nitrogen fixation (NIF), and the cytosolic iron-sulfur cluster assembly (CIA) systems. The best characterized Fe–S cluster biogenesis pathway is the ISC system, which requires the HscA/HscB [also known as heat shock protein 70 (Hsp70)/J-protein] chaperone/co-chaperone ATPase complex. Like most NTPases, these chaperone/co-chaperone proteins use nucleoside triphosphate binding and hydrolysis to regulate the transfer of a newly synthesized Fe–S cluster from a scaffold protein to an apo-recipient.^61,62^ The NIF system uses analogous chaperone/co-chaperone proteins but also requires an ATPase to deliver homocitrate-bound molybdenum during the process of assembling the FeMo-cofactor of nitrogenase; the roles of ATPases in nitrogenase maturation have been recently reviewed.^5,63^ In the SUF pathway, the ATP-binding cassette (ABC)-type ATPase SufC is necessary for Fe–S cluster formation.^64^ SufB, which accepts sulfur, and SufD, which has been proposed to play a role in iron acquisition, have been shown to interact with each other *in vitro*.^64,65^ Along with SufB and SufD, deletions of SufC abolish SUF function *in vivo. In vitro*, the SufBC_2_D complex can serve as a scaffold for de novo Fe–S cluster biogenesis.^66^ Recent structural studies of the SufBC_2_D scaffold provide insight into how ATP binding to SufC may promote conformational changes that are necessary for the formation of the cluster assembly site; however, a detailed mechanistic understanding of the ATPase cycle for cluster formation is not yet available.^64,67^ Finally, the CIA pathway uses a scaffold ATPase to assemble new Fe–S clusters; ATPase activity is required for both [2Fe–2S] cluster acquisition and transfer of the fully formed [4Fe–4S] cluster to the apo-client in the cytosol or nucleus.^68–70^ Although many of the participants of these pathways have been identified, their biochemical characterizations are limited.^62^ Recently, Perlstein and co-workers have provided a biochemical roadmap for exploring the roles of ATP-binding sites and ATPase activity for proteins involved in Fe–S cluster biogenesis.^71^

## Nucleotide specificity of NTPase metallochaperones can be challenging to establish

Not all NTPase metallochaperones fall clearly into a GTPase subgroup or an ATPase subgroup. Although the better studied HypBs are GTPases, some organisms have versions of HypB that are ATPases.^72,73^ The HypB from the archaea *Thermococcus kodakarenis* is a P-loop ATPase containing the same overall fold as the characterized GTPase HypBs. Despite less biochemical characterization, there is evidence in some organisms that the HypB ATPases are functional homologs that replace the HypB GTPases, with their activity also regulated by Ni^2+^-binding, ATP hydrolysis, and interactions with HypA.^73^ Also, the Feo iron transport system appears to utilize an NTPase called FeoB for which the nucleotide specificity has been unclear.^74–80^ The Feo system is a dedicated Fe^2+^ ion transport system that is widely present in the archaeal and bacterial domains of life. It is composed of FeoA and FeoC, cytosolic proteins, and the transmembrane iron permease, FeoB. FeoB from the human pathogen *H. pylori* is thought to be an ATPase due to impaired Fe^2+^ ion transport when whole cells are treated with known inhibitors of ATP synthesis or hydrolysis.^81^ In contrast, FeoB from *E. coli* is believed to be a GTPase because no ATP binding was observed *in vitro*.^82^ The characterization of the FeoB from *Vibrio cholerae* showed both low intrinsic GTPase and ATPase activity that was not stimulated by any known factors.^78,79^ Research by Kim and coworkers on various pathogenic bacterial FeoBs suggests that there are two classes of FeoBs: sole GTPases and promiscuous NTPases.^80^

FeoB, and also HypB, highlight the difficulty in establishing nucleotide specificity for NTPase metallochaperones. It is clear in the case of HypB, and likely in the case of FeoB, that different organisms employ different NTPases (GTPases or ATPases). The reason(s) for this variation are not well established. However, it also seems to be the case that some metallochaperone systems have no variation; for example, there are no known MeaB metallochaperones that are ATPases. Yet in other systems, NTPases appear to be employed that are promiscuous. It is not known if this promiscuity is an *in vitro* or *in vivo* feature. Given that most NTPase metallochaperones are poor NTP hydrolases without their target protein and/or without the appropriate metal ion bound, one must be careful about drawing conclusions, both favorable and unfavorable, from low levels of NTP hydrolysis activity. Additional studies may reveal that all NTPases are specific under the right set of conditions and/or that more GTPases have ATPases cousins. A MeaB that is an ATPase may be discovered, for example. It is currently not possible to predict nucleotide specificity from protein sequences, but more structural data will likely improve the prediction possibilities.

## Metal specificity of NTPase metallochaperones can be challenging to establish

An important role of metallochaperones is to make sure that the correct metal ion is inserted into the correct apo-metalloprotein target, raising the question of how the metal specificity of metallochaperones is established.^83^ The biological challenge of correct protein metalation has been recently reviewed.^84^ Briefly, one important factor in metalation is cellular metal availability, which is thought to be opposite of the Irving–Williams series, Mg^2+^< Mn^2+^ < Fe^2+^ < Co^2+^ < Ni^2+^ <Cu^2+^ > Zn^2+^, with the weaker binding metals such as Mg^2+^, Mn^2+^, and Fe^2+^ more widely available and the tighter binding metals such as Ni^2+^, Zn^2+^, and Cu^2+^ less available.^85–87^ The reducing/oxidizing environment in the cell is also a consideration in terms of availability. For example, copper ions in the cell are thought to be in the Cu^1+^, rather than Cu^2+^ state given the reducing environment of the cytoplasm. Availability further depends on the ability of the organism to uptake metal ions, especially trace metals. In addition to availability, another factor affecting specificity is the metallochaperone's affinity for different metal ions, a feature of the protein that can be modulated by GTP binding or hydrolysis. All of this information points to the fact that establishing the metal specificity of a metallochaperone can be challenging.^88^

## Establishing metal specificity for the GTPase metallochaperone CobW

The observation that *cobW* gene disruption in *Pseudomonas denitifricans* impairs aerobic cobalamin biosynthesis led to the proposal that CobW is a Co^2+^-dependent metallochaperone.^29,30^ To test this proposal, affinities were measured for Co^2+^ and other metal ions for the GTPase-dependent CobW from *Rhodobacter capsulatus* in the presence and absence of nucleotide effectors.^89^ Only weak interactions between CobW and Co^2+^ ions were observed in the absence of nucleotides. The addition of GTP or less hydrolyzable analogs promotes the tight coordination of two Co^2+^ ions in two different binding sites with different affinities. However, if Mg^2+^ ions are also present at physiological concentrations, the coordination of a Co^2+^ ion is observed in only one binding site. The other site is occupied by a Mg^2+^ ion. It is thought that the Mg^2+^ ion binds first in the weak affinity metal-binding site, ordering the second binding site for the high affinity binding of a Co^2+^ ion. When GDP is present instead of GTP, Co^2+^ binds CobW with a 1000-fold weaker affinity, indicating that an intact γ-phosphate is required for tight binding. The presence of bound GDP causes CobW to release the Co^2+^, further tying the nucleotide state to the metalation state of CobW. Finally, when compared to other first row transition metals, Mg^2+^GTP-CobW binds Zn^2+^ and Cu^1+^ more tightly;^89^ however, using the idealized pool of bioavailable metals^90^ and the free energy change for metal binding, calculations indicated that *in vivo* over 90% of Mg^2+^GTP-CobW would be bound with Co^2+^, as compared to less than 10% bound with Zn^2+^ due to the greater favorable free energy change for Co^2+^ binding.^89^ This recent work firmly establishes Co^2+^ as the cognate metal for CobW, supporting its known involvement in cobalamin biosynthesis. Additionally, these studies confirm the presumed role of GTP binding and hydrolysis for metalation of the metallochaperone, like other G3E P-loop GTPases.^89^

## Final thoughts and future directions

The roles of NTPase metallochaperones continue to expand with new functions being established. However, there is much more work to be done. The COG0523 subfamily of G3E P-loop GTPases, for example, represents a poorly understood class of GTPase metallochaperones that are believed to bind and insert transition metals.^50^ Many of these putative GTPases are uncharacterized and their target client proteins are unknown. Bioinformatic studies of the gene clusters containing these uncharacterized GTPases may allow for predictions of the target client proteins. Predictions should be followed by biochemical characterizations exploring NTPase:target protein interactions, NTP specificity, and metal ion specificity. The latter can potentially be aided by the application of methods/calculations used in the CobW studies described earlier.^89^

Genomic initiatives and the wealth of sequence information that they generate are leading to proposals of putative NTPase metallochaperones outside of the COG0523 family. For example, the protein MutS is well known for its involvement in DNA repair processes;^91^ however, bioinformatics has revealed sequences of MutS-like proteins in operons associated with adenosylcobalamin-dependent enzymes, leading to a proposal that MutS variants might play a role in ATP-dependent metalloprotein complex assembly.^92^ We hypothesize that there are likely other examples of ATPase families whose distant cousins are involved in diverse biological processes. We are excited about how modern bioinformatics methods combined with genomic data will undoubtedly expand the NTPase metallochaperone field in the near future.

Among the already characterized NTPase metallochaperones, numerous questions remain. For example, it is often unclear whether the complete complement of stimulatory factors that increase NTP hydrolysis has been identified; *is another protein involved or maybe another metal ion*? Molecular mechanistic questions are also prevalent*; what exactly is the function of the nucleotide-state-dependent conformational change, and what type of conformational change occurs*? Structural information on GTPase metallochaperones is fairly limited (Table 2), and even when structural methods have allowed for the capture of more than one nucleotide-bound state, it is often the case that crystal lattice contacts have prevented the conformational change from occurring.^36^ Additionally, the absence of the target protein or other stimulatory factors may hinder the nucleotide-state-dependent conformational change of the NTPase from being fully realized.^33^ In other words, obtaining the requisite structural snapshots of NTPases to understand their molecular mechanisms is not trivial, and for the most part, these structural data are missing for NTPase metallochaperones, leaving molecular mechanistic questions unanswered. For example, there is more to learn about the steps required to transfer nickel from the GTPase HypB to its partner metallochaperone HypA to complete the [NiFe]-hydrogenase active site. The extent of the conformational changes that occur in HypB due to GTP binding and hydrolysis has not been visualized, limiting the molecular understanding of the mechanism.^45,93^ In the maturation of the adenosylcobalamin-dependent methylmalonyl-CoA mutase, the GTPase MMAA (MeaB in bacteria) is required for adenosylcobalamin insertion but does not bind the cofactor directly.^24,35,46–49,94^ Although there are a number of structures available of MMAA/MeaB in various nucleotide-bound states,^33–35^ and even a structure of a MeaB-fusion protein in which the chaperone domain is covalently attached to the target enzyme,^36^ the molecular basis by which MMAA/MeaB facilitates adenosylcobalamin delivery is still in debate. Currently, none of the structural rearrangements observed explain the molecular basis of methylmalonyl-CoA mutase maturation.^34,36^ Cryo-electron microscopy (cryo-EM) represents a promising new direction for the obtainment of these requisite structural data as this method allows for structures of protein:protein complexes to be obtained and for multiple conformations of proteins to be more readily visualized. Although the NTPases by themselves are too small for cryo-EM, the protein:protein complexes involved in metalloprotein maturation should be sufficiently large,^95^ and ultimately, it is the structures of protein:protein complexes that are needed for a molecular understanding. Thus, the resolution revolution of cryo-EM, i.e. the revolutionary ability to determine near-atomic resolution protein structures by cryo-EM, represents an exciting prospect moving forward for the determination of structures of metalloprotein maturation machineries.

An improved understanding of metalloenzyme maturation has several possible industrial applications. For example, nitrogenases are attractive as environmentally friendly alternatives to the industrial Haber–Bosch process, which is estimated to use ∼1% of the world's energy. This alternative solution would be even more attractive if nitrogenases could be prepared at high levels with their complex metallocofactors correctly inserted (Fig. 1). Hydrogenases are appealing for use in biofuel cells, and CODHs for fixation of the greenhouse gas carbon dioxide. Again, these applications require that the metalloproteins be produced in high yield, which necessitates an understanding of cofactor biogenesis/delivery. Additionally, knowledge of metalloenzyme maturation processes could be exploited to deliver synthetic metallocofactors with altered reactivity to apo target enzymes.

Regarding human health, understanding the molecular mechanisms of NTPase metallochaperones may provide novel solutions for therapeutics and treatments.^11,17,96–98^ For example, methylmalonic aciduria, an inborn error of metabolism, is caused by deletions of or mutations to any of the numerous proteins that transport and/or insert adenosylcobalamin into methylmalonyl-CoA mutase, including MMAA. Since MMAA does not interact with the cofactor directly, understanding how MMAA uses GTP binding and hydrolysis to perform its gatekeeping role could provide a novel therapeutic strategy. Additionally, NTPase metallochaperones thought to be involved in virulence, such as FeoB and UreG in *H. pylori*, are additional drug targets.^13,81^

In closing, we dedicate this minireview to the memory of Professor Deborah Zamble, who was a leader in the field of nickel enzymes and contributed to much of the research described earlier. Deborah left us too soon, and the work she started is not yet complete. There are many fascinating aspects of NTPase metallochaperones awaiting discovery and applications of metalloenzymes to be pursued. For young scientists looking to make a mark, metalloprotein maturation processes are a ripe area for discovery.

## Data Availability

No new data were generated or analyzed in support of this research.
